# “Don’t Blast”: blast-in-place (BiP) operations of dumped World War munitions in the oceans significantly increase hazards to the environment and the human seafood consumer

**DOI:** 10.1007/s00204-020-02743-0

**Published:** 2020-04-18

**Authors:** Edmund Maser, Jennifer S. Strehse

**Affiliations:** Institute of Toxicology and Pharmacology for Natural Scientists, University Medical School Schleswig-Holstein, Campus Kiel, Brunswiker Str. 10, 24105 Kiel, Germany

**Keywords:** Dumped munitions, Marine environment, Biomonitoring, Blue mussels, Blast-in-place operations, Marine food chain, Risk assessment

## Abstract

The seas worldwide are threatened by a “new” source of pollution: millions of tons of all kind of warfare material have been dumped intentionally after World War I and II, in addition to mine barriers, failed detonations as well as shot down military planes and sunken ship wrecks carrying munitions. For example, in the German parts of the North and Baltic Sea approximately 1.6 million metric tons of toxic conventional explosives (TNT and others) and more than 5000 metric tons of chemical weapons are present. Such unexploded ordnance (UXO) constitutes a direct risk of detonation with increased human access (fisheries, water sports, cable constructions, wind farms and pipelines). Moreover, after more than 70 years of resting on the seabed, the metal shells of these munitions items corrode, such that chemicals leak out and distribute in the marine environment. Explosive chemicals such as TNT and its derivatives are known for their toxicity and carcinogenicity. In order not to endanger today's shipping traffic or the installation of pipelines and offshore plants by uncontrolled explosions, controlled blast-in-place (BiP) operations of these dangerous relics is a common practice worldwide. However, blast-in-place methods of in situ munitions disposal often result in incomplete (low-order) detonation, leaving substantial quantities of the explosive material in the environment. In the present free field investigation, we placed mussels (*Mytilus *spp.) as a biomonitoring system in an area of the Baltic Sea where BiP operations took place and where, by visual inspections by scientific divers, smaller and larger pieces of munitions-related materials were scattered on the seafloor. After recovery, the mussels were transferred to our laboratory and analyzed for TNT and its derivatives via gas chromatography and mass spectroscopy. Our data unequivocally demonstrate that low-order BiP operations of dumped munitions in the sea lead to multiple increases in the concentration of TNT and its metabolites in the mussels when compared to similar studies at corroding but still encased mines. For this reason, we explicitly criticize BiP operations because of the resulting environmental hazards, which can ultimately even endanger human seafood consumers.

## Introduction

The seas worldwide are threatened by a “new” source of pollution: Millions of tons of all kind of warfare material, mainly resulting from war activities during the last century, contaminate the oceans, especially coastal regions (Beddington and Kinloch 2005; Carton and Jagusiewicz 2011). The majority of these munitions were the result of dumping activities after the First and Second World War, but sunken underwater mine barriers, unexploded ordnances and wrecks of military planes and war ships carrying munitions are contributing to this problem as well. In particular, coastal sites in Europe, North America and the southwest Pacific are heavily affected (Monfils et al. 2006).

For example, it has been estimated that the German parts of the North and Baltic Sea alone contain approximately 1.6 million metric tons of conventional explosives (TNT and others) and more than 5000 m tons of chemical weapons (Böttcher et al. 2011; Nehring 2005) as relicts from the two World Wars. In addition, an unknown amount of modern ammunition from the Federal German Navy, the former National People’s Army of the German Democratic Republic, NATO and Soviet Navy activities are present in German waters (Nehring 2008).

Relic underwater conventional and chemical munitions represent multiple hazards to human activities. As the precarious and first danger, uncontrolled self-detonations by ship traffic (civil and military), fisheries, water sports, cable constructions, wind farms and pipelines were primarily feared. Even more, after long periods of resting under water, the sensitivity of the explosive materials increases due to deterioration of stabilizing components or recrystallization (Pfeiffer 2012).

Recently discovered and potentially serious is the fact that, after more than 70 years of laying on the seafloor, the metal shells of the dumped munition items are corroded, and their toxic compounds leak out and get distributed in the marine environment (Appel et al. 2018; Beck et al. 2019; Böttcher et al. 2011; Strehse et al. 2017). Explosive chemicals like TNT and its derivatives are known for their toxicity and carcinogenicity (Bolt et al. 2006; Sabbioni and Rumler 2007) and pose a great threat to the marine environment (Talmage et al. 1999). Aquarium and free field toxicity studies with freshwater organisms and several marine species suggest that the levels of explosive chemicals at munitions-contaminated sites are unlikely to exhibit acute toxicity on biota due to slow dissolution and extensive dilution. However, there is growing evidence that munition-related chemicals can cause sublethal and chronic effects in aquatic biota, especially in benthic and epifaunal organisms (Ek et al. 2006, 2008; Juhasz and Naidu 2007; Lotufo et al. 2013; Rosen and Lotufo 2007; Talmage et al. 1999).

In spite of the considerable amounts of leaking warfare material, little is known about the potential effects on the marine environment and marine organisms. On the one hand, it is not clear how well toxicological laboratory experiments represent natural communities in the marine environment (Ek et al. 2008; Nipper et al. 2001, 2002). On the other hand, determining the occurrence of munitions constituents in natural ecosystems is still an analytical challenge. Another hazard in addition to the acute risk of detonation and chronic threat to the marine environment is that leaking explosive chemicals may enter the marine food chain and directly affect human health upon consuming contaminated seafood (Ek et al. 2006, 2007, 2008).

To prevent endangering today's shipping traffic or the installation of pipelines and offshore plants by uncontrolled and unforeseen explosions, controlled blast-in-place (BiP) operations of these dangerous World War relicts are a common practice worldwide. For this purpose, contact donor charges are attached manually that initiate detonation of these munition bodies. However, BiP operations of in situ munitions disposal (Koschinski and Kock 2009) often result in incomplete (low-order) detonation, leaving substantial quantities of the explosive material in the environment (Kalderis et al. 2011; NATO 2010; Pfeiffer 2007).

The German Baltic Sea is a marine area with large amounts of munitions deposited. For example, “Kolberger Heide” as a part of the Kiel Bight served as a dumping ground with at least 8000 torpedo heads and 10,000 mines among other items such as ground mines and moored contact mines that were dumped after World War II (Böttcher et al. 2011; Nehring 2005, 2008). “Kolberger Heide” also served for “removal” of old munitions by BiPs, in case these munitions items were considered to be an acute threat. If located elsewhere in the Kiel Bight, munition items were also pulled to BiP places within Kolberger Heide before blasting. For example, numerous 250–500 kg mines were intentionally detonated underwater via BiP in 2009 (Haas and Thieme 1996; Pfeiffer 2009). Unfortunately, these low-order detonation left substantial quantities of intact explosive material on the seafloor. A visual inspection by scientific divers proved that, although the acute danger from uncontrolled explosions was eliminated, a large number of large and small chunks of explosive material had been distributed in this area (Appel et al. 2018; Beck et al. 2019; Strehse et al. 2017).

As part of a national consortium that was funded by governmental sources, we have established the mussel *Mytilus* spp. as a biomonitoring system to determine explosive chemical contaminations leaking from corroding mines (Appel et al. 2018) or dissolving from a large chunk (half meter scale) of hexanite (German “Schiesswolle” consisting of 45–67% TNT, 5–24% hexanitrodiphenylamine and 16–25% aluminium powder) (Strehse et al. 2017) into the marine environment. With this system, we could unequivocally show that explosive residues enter the marine biota in the vicinity of dumped munitions.

The current study was conducted to uncover the risks of BIPs, for the environment as well as for the human seafood consumer. Our field biomonitoring approach included exposure of blue mussels by scientific divers to either corroding mines or directly to a free-lying chunk of hexanite resulting from a BIP operation. Finally, mussels were exposed on the seafloor in the same area, but with multiple explosive pieces scattered on the seafloor after BiP operations. Mussel tissues were then analyzed in the laboratory by gas chromatography/mass spectrometry for TNT and its derivatives. The data obtained were then used to carry out a toxicological risk assessment.

According to our biomonitoring findings in a BiP area of the German Baltic Sea, we explicitly criticize blast-in-place operations because of the resulting environmental hazards, which can ultimately even endanger human seafood consumers.

## Materials and methods

### The study site: Kolberger Heide in the Baltic Sea near Kiel

**Fig. 1:**
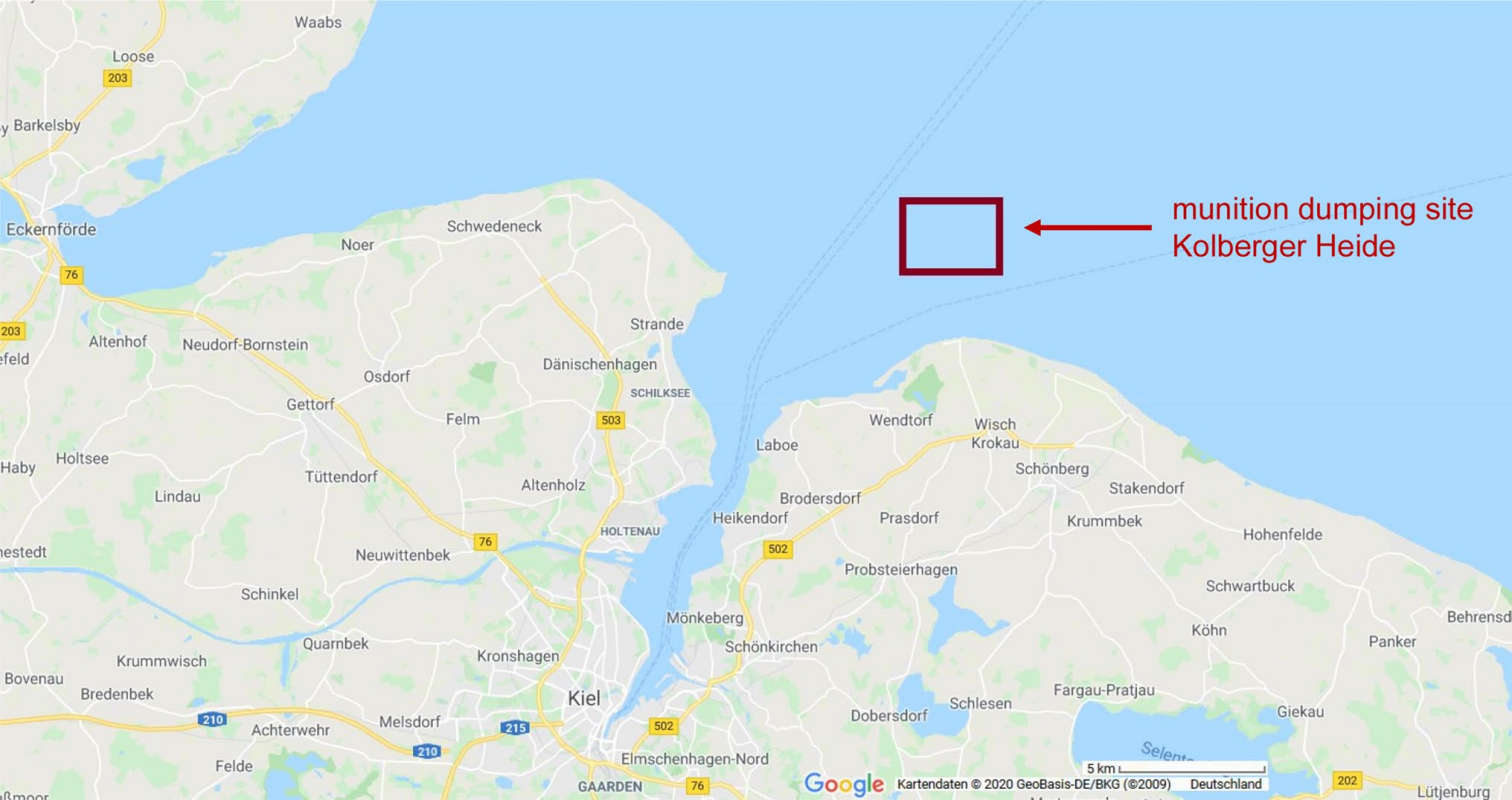
The study area Kolberger Heide. Kolberger Heide is a section of the western Kiel Bight at the entrance to Kiel Fjord, Germany, with a size of approximately 1260 km^2^, located in a distance of three to five nautical miles to the shoreline. After World War II, it has been used as an area for dumping munitions (red insert). Source: https://www.google.de/maps/ (color figure online)

The Kolberger Heide, located in the southwest of the Baltic Sea near Kiel Fjord (at approximately 54.46° N and 10.34° E), is one of the former dumping sites of World War II munitions relicts both from German and British sources (Fig. 1). The area has a size of approximately 1.260 ha, is 10–15 m deep and is located at a distance of three to five nautical miles to the shoreline. According to estimates, it contains up to 10,000 mines of different types, 8000 torpedo heads, many depth charges, ground mines and moored mines (Nehring 2008). There are also indications of scattered naval artillery shells among the originally dumped mines. Kolberger Heide was used for “storage” of old munitions, in case these munition items were considered to be an acute threat. For example, 110 British ground mines that have been found close to navigational routes since 2012 were translocated into this area by the state explosive ordnance disposal service (Blano et al. 2012, 2011). The area also served for BiP operations, such as for example numerous 250–500 kg mines that were intentionally detonated underwater by military explosive ordnance disposal experts between 2009 and 2012 (Haas and Thieme 1996; Pfeiffer 2009).

**Fig. 2:**
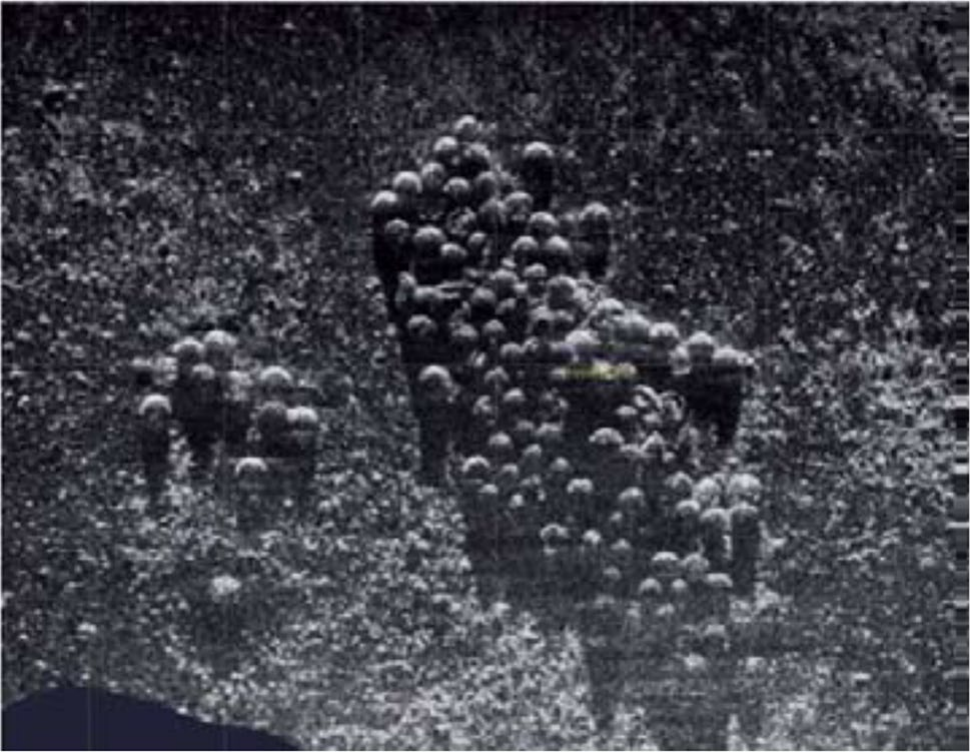
Hi-SAS-Sonar image of some 70 moored mines from the Second World War in study sub-site 1 in the Kolberger Heide. Source: Deutsche Marine 2012

Unfortunately, these low-order detonations left substantial quantities of intact explosive material scattered on the seafloor. A visual inspection by scientific divers proved that multiple larger solids (half mete scale) and smaller pieces (10–30 cm scale) of explosive material had been distributed in this area (Appel et al. 2018; Beck et al. 2019; Strehse et al. 2017). Within the Kolberger Heide site, munition items are not only widely scattered, but are also clustered, such as in a large pile of approximately 70 sea mines (Fig. 2).

**Fig. 3:**
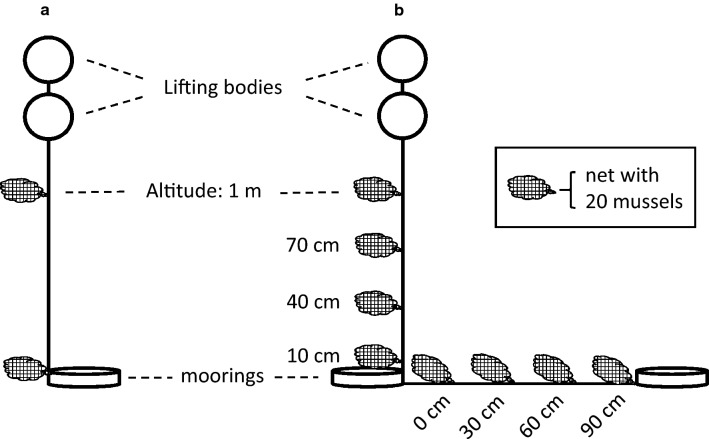
Construction scheme of the moorings positioned at Kolberger Heide. **a** At study sub-sites 1 and 2; **b** at study sub-site 3

Overall, the heterogeneous distribution and diversity of intact or corroding, but still encased munitions items on the one side, as well as free-lying larger solids and smaller pieces of munition materials on the other side, provided us a unique opportunity to study the consequences of BiP operations in the Kolberger Heide (Fig. 3). Moreover, based on our free field data we could perform a preliminary toxicological risk assessment regarding the human seafood consumer.

### Study sub-site 1: six moorings at corroding mines

**Fig. 4:**
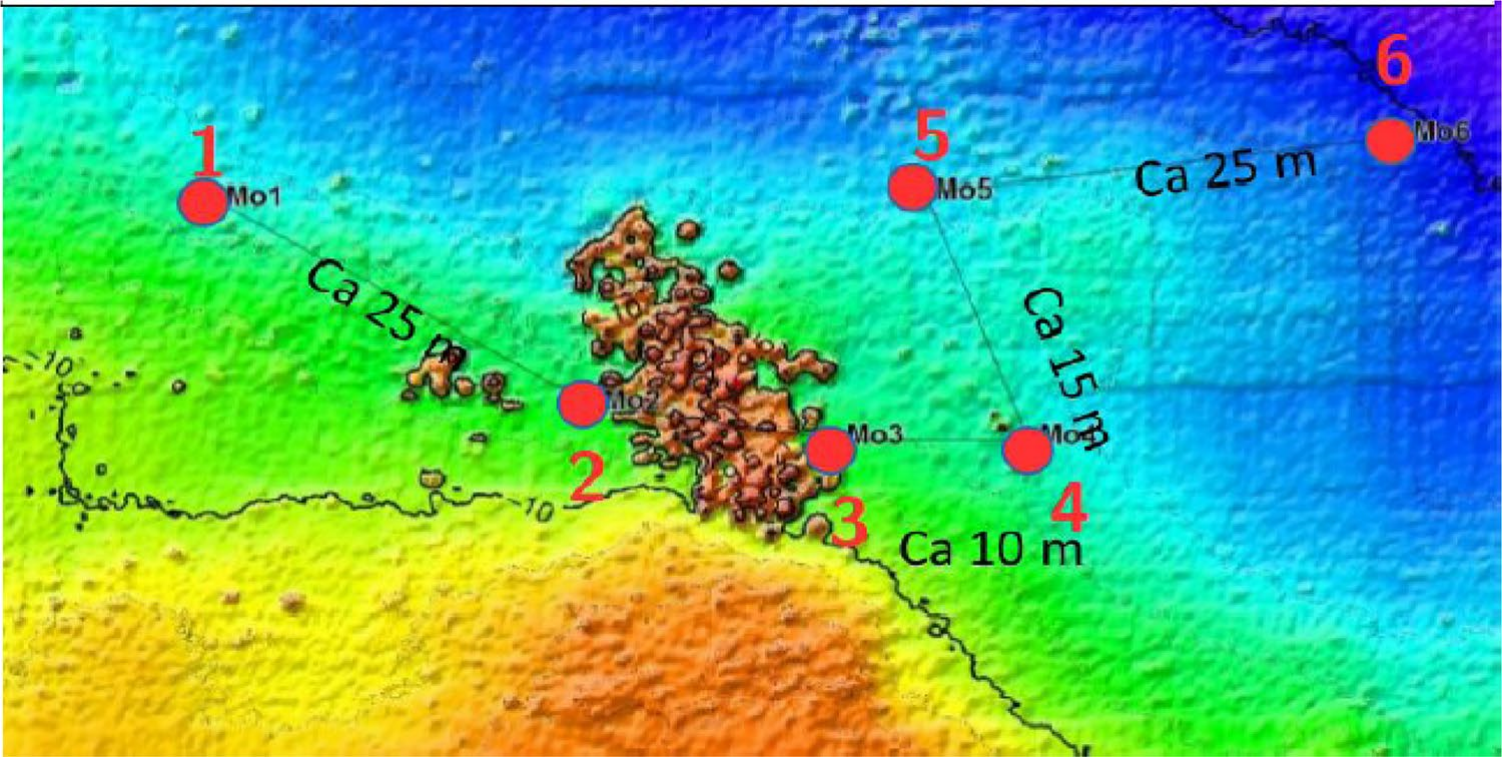
Study sub-site 1. Position of Moorings No. 1–6 (red dots) placed around the mine mount consisting of approximately 70 moored mines. © M. Kampmeier/Geomar (color figure online)

Mussels for exposure studies were transplanted by scientific divers in direct vicinity to corroding mines of a mine mound consisting of approximately 70 items from World War II (Fig. 4). Six moorings (Moorings No. 1–6) with two mussel bags each (see Fig. 3a), one directly on the ground (P0) and one 1 m above ground level (P1), had been placed at different distances (closest distance 0 m) from the mine mound. Each mussel bag contained 20 mussels with a size of approximately 48.1 mm ± 9.6 mm in length. The exposure period amounted to 106 days. Sampling was performed by the explosive ordnance disposal unit (state office of criminal investigation) and/or Kiel University research divers. After recovery, the mussels were immediately placed on dry ice and stored at − 80 °C until preparation for GC–MS/MS analysis. More details are described in Appel et al. (2018).

### Study sub-site 2: one mooring at a chunk of unexploded hexanite

**Fig. 5:**
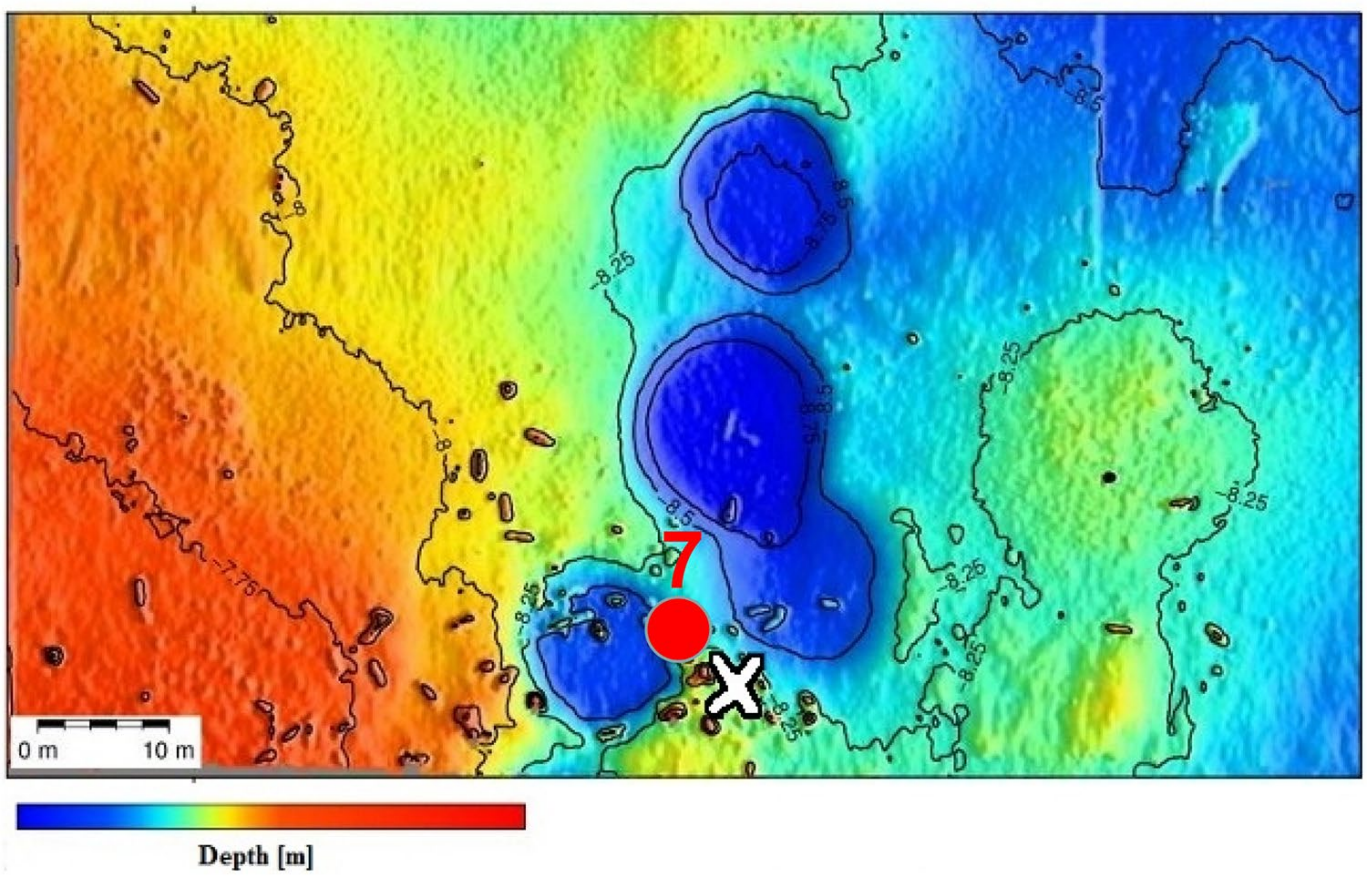
Study sub-sites 2 and 3. Position of Mooring No. 7 (red dot); position of the vertical and horizontal lines (indicated by a white cross) near craters caused by controlled blast in place (BiP) operations in the southern part of the study area Kolberger Heide. © M. Kampmeier/Geomar (color figure online)

Another mooring (Mooring No. 7) (Fig. 3a) was placed directly at a chunk (half meter scale) of explosive material (hexanite = German “Schiesswolle”) close to a blast crater site (Fig. 5). As with moorings No. 1–6, this Mooring No. 7 was equipped with the same arrangement of two mussel bags and the same number of mussels (see above). The exposure period was 93 days. More details are described in Strehse et al. (2017).

### Study sub-site 3: horizontal line over scattered smaller munition solids

To evaluate the entry of explosive chemicals into the mussels in an area with larger and smaller pieces (5–30 cm in diameter) scattered on the seafloor, four mussel bags (each containing 10 mussels of length and size described above) were deposited in 30 cm intervals horizontally on the seafloor and vertically in the water column (Fig. 3b; Fig. 5). The exposure period amounted to 106 days.

### Chemicals

2,4,6-Trinitrotoluene (TNT), 2-amino-4,6-dinitrotoluene (2-ADNT), 4-amino-2,6-dinitrotoluene (4-ADNT) and 2,4-diamino-6-nitrotoluene (2,4-DA-6-NT) were purchased from AccuStandard (New Haven, CT, USA) with a chemical purity of 100% for all compounds. Gradient grade acetonitrile was purchased from Th. Geyer GmbH & Co. KG (Renningen, Germany).

### The blue mussel *Mytilus *spp.

Blue mussels (*Mytilus* spp.) were obtained from a commercial mussel farm located in Kiel Fjord. Mussels were transported in cooled insulation boxes with seawater to the shipping vessel and transferred to the study site Kolberger Heide or were immediately stored deep frozen as respective unexposed controls.

### Sample preparation

Extraction and analysis of TNT, 2-ADNT, 4-ADNT and 2,4-DANT was carried out as described previously (Strehse et al. 2017). In brief, mussels (*n* = 3–6 individuals) were thawed, and whole meat was placed in 50 mL polypropylene tubes, and homogenized using a T25 Ultra-Turrax (Ika Works Inc., Staufen im Breisgau, Germany). Tissues were aliquoted into 1.0 g portions in 50 mL polypropylene tubes, and 5 mL gradient grade acetonitrile (Th. Geyer GmbH&Co. kg, Renningen, Germany) was added per tube. Each sample was mixed for 1 min using a VF2 vortex mixer (Ika Works Inc., Staufen im Breisgau, Germany). Tubes were centrifuged for 5 min at 4100 rpm (20 °C) with a Heraeus Megafuge 11R centrifuge (Thermo Fisher Scientific Inc., Waltham, MA, USA). Supernatants were decanted and filled with acetonitrile to a total volume of 10.0 mL, followed by GC/MS-MS analysis.

### GC/MS–MS analysis

For GC/MS–MS analysis 1 µl of the samples was injected with a splitless liner in a Trace 1310 Gas Chromatograph (Thermo Fisher Scientific Inc., Waltham, MA, USA) and analytes were separated on a TG-5SILMS GC column (15 m × 0.25 mm × 0.25 μm) (Thermo Fisher Scientific Inc., Waltham, MA, USA). Helium as carrier gas was used with a carrier flow rate of 1.5 ml/min and a split flow rate of 20 ml/min. The oven temperature program was as follows: 1 min at 120 °C, heating to 220 °C with a heating rate of 30 °C/min, heating to 300 °C with a heating rate of 50 °C/min up to 300 °C, 300 °C held for 0.5 min. The retention times for TNT, 4-ADNT, 2-ADNT and 2,4-DA-6-NT were 3.50 min, 4.32 min, 4.50 min and 4.64 min, respectively. Eluted analytes were ionized with electron ionization (EI) or negative chemical ionization (CI) with methane as the reagent gas and analyzed with a TSQ 8000 EVO Triple Quadrupole Mass Spectrometer (Thermo Fisher Scientific Inc., Waltham, MA, USA) in MRM-mode.

### Data analysis

Measured values are expressed as mean ± standard deviation.

## Results

### Biomonitoring with blue mussels (*Mytilus *spp.)

Here, we report biomonitoring data from mussel samples exposed at three different study sub-sites within the Kolberger Heide: (1) in the direct vicinity to a large pile of corroding discarded sea mines, (2) a free-lying chunk of explosive material, and (3) smaller pieces of hexanite, the latter two items resulting from low-order detonation during blow-in-place (BIP) methods of in situ munitions disposal Federal State of Schleswig Holstein (Germany). Mussels were manually exposed at the three sub-sites by divers, collected after 3 month periods and processed using a slight modification of published methods (Appel et al. 2018; Strehse et al. 2017).

Results derived in study sub-sites 1 and 2 have been, in part, already presented in Appel et al. (2018) and Strehse et al. (2017), and are given here for comparison with study sub-site 3. The detection limit for all compounds measured by GC/MS-MS and electronionization (EI) was 1.5 ng/g mussel wet weight. The detection limit with chemical ionization (CI) was about threefold lower than with EI. For all measurements with exposed mussels, we used electron ionization. For the non-exposed control mussels, we used both electron and chemical ionization. No TNT, 2-ADNT, 4-ADNT or 2,4-DA-6-NT was detectable in the non-exposed control mussels.

### Study sub-site 1: six moorings at corroding mines

**Fig. 6:**
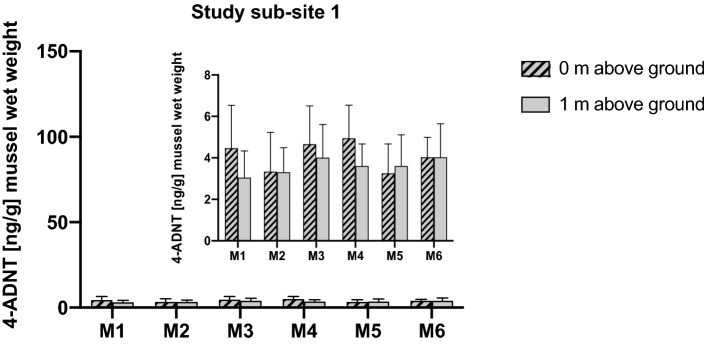
Results from study sub-site 1. mean values (± SD) of 4-amino-2,6-dinitrotoluene (4-ADNT) measured in the tissues of *Mytilus* spp. (*n* = 8 from P0, *n* = 7 from P1) exposed at a mound of approximately 70 corroding mines in the Kolberger Heide. Of note: *Y*-axis scale 0–150 ng/g is given for comparison with other sub-sites. The insert provides a better view on the data

In mussels exposed in direct vicinity to the corroding mines, only 4-ADNT, a metabolite of TNT, was found. In neither mussel of the six moorings (Mooring No. 1–6) arranged around the mine mount 2-ADNT, 2,4-DA-6-NT or TNT itself could be detected. Overall, 4-ADNT concentrations were low, ranging from 3.31 ± 1.17 to 4.94 ± 1.29 ng/g (mussel wet weight) and did not differ significantly with regard to the distance to the mine mount or regarding the position of the mussels on the mooring (1 m above or directly on the ground) (Fig. 6).

### Study sub-site 2: one mooring at a chunk of unexploded hexanite

**Fig. 7:**
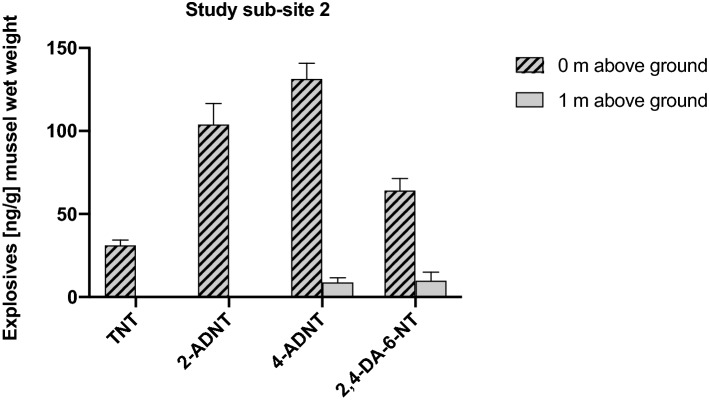
Results from study sub-site 2. mean values (± SD) of TNT, 2-amino-4,6-dinitrotoluene, 4-amino-2,6-dinitrotoluene and 2,4-diamino-6-nitrotoluene (2,4-DA-6-NT) measured in the tissues of *Mytilus* spp. (*n* = 8 from P0, *n* = 7 from P1) exposed at a free-lying chunk (50 cm scale) of hexanite near BiP craters in the Kolberger Heide

In mussels of Mooring No. 7, which was placed directly at a chunk (50 cm diameter in size) of explosive material (hexanite) close to a blast crater site, the TNT metabolites 2-ADNT, 4-ADNT and 2,4-DA-6-NT as well as TNT itself were detected. The average concentrations in mussels exposed at mooring No. 7 directly on the ground were: 2-ADNT = 103.75 ± 12.77 ng/g, 4-ADNT = 131.31 ± 9.53 ng/g, 2,4-DA-6-NT = 64.03 ± 7.34 ng/g and TNT = 31.04 ± 3.26 ng/g mussel wet weight, respectively. In mussels placed 1 m above the ground neither TNT nor 2-ADNT could be detected, but 4-ADNT and 2,4-DA-6-NT have been determined with average concentrations of 8.71 ± 2.88 ng/g and 9.84 ± 5.15 ng/g mussel wet weight, respectively (Fig. 7).

### Study sub-site 3: horizontal and vertical lines over scattered smaller pieces of hexanite

**Fig. 8:**
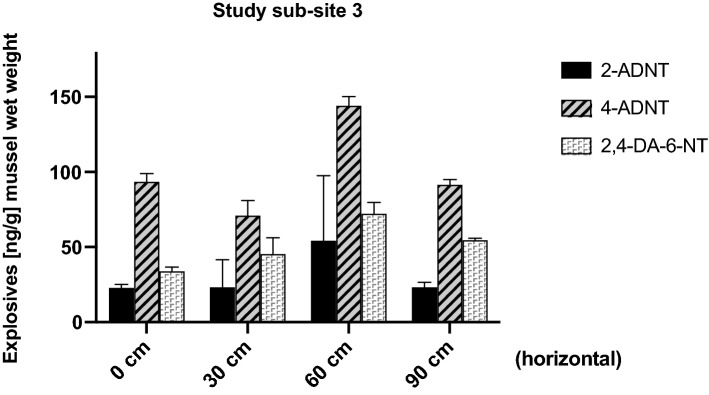
Results from study sub-site 3. Horizontal line: mean values (± SD) of 2-amino-4,6-dinitrotoluene (2-ADNT), 4-amino-2,6-dinitrotoluene (4-ADNT) and 2,4-diamino-6-nitrotoluene (2,4-DA-6-NT) measured in the tissues of *Mytilus* spp. (*n* = 3) exposed horizontally in a region of scattered solids (10–30 cm scale) of hexanite near BiP craters in the Kolberger Heide

Mussels exposed to explosives horizontally in 30 cm intervals in a region with larger and smaller pieces of explosive material scattered on the seafloor showed the highest concentrations of TNT metabolites (Fig. 8). Again, 4-ADNT appeared to be the most abundant explosive compound found in all samples, ranging between 70 and 145 ng/g mussel wet weight. 2-ADNT varied between 22 and 55 ng/g mussel wet weight, while 2,4-DA-6-NT ranged between 33 and 73 ng/g mussel wet weight. No TNT was found. Apparently, compared to corroding (but still encased) munitions, the free-lying explosive fragments resting on the seabed, either in the form of larger chunks or smaller pieces, led to an approximately 30–50-fold increased entry and concentration of explosive chemicals within the exposed mussels (Fig. 8).

**Fig. 9:**
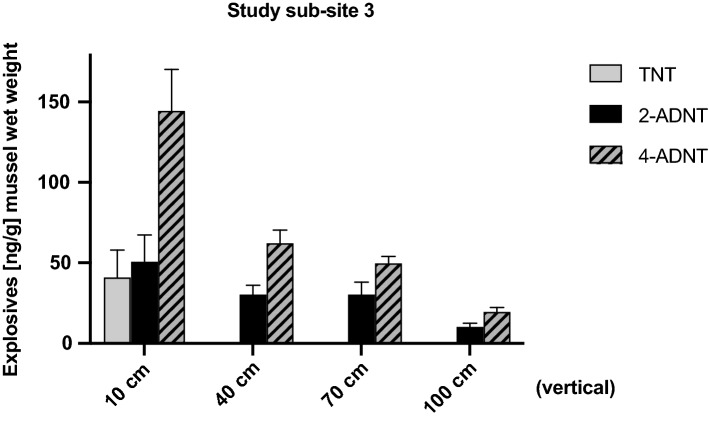
Study sub-site 3. Vertical line: mean values (± SD) of TNT, 2-amino-4,6-dinitrotoluene (2-ADNT) and 4-amino-2,6-dinitrotoluene (4-ADNT) measured in the tissues of *Mytilus* spp. (*n* = 3–5) exposed vertically in a region of scattered solids (10–30 cm scale) of hexanite near BiP craters in the Kolberger Heide

Likewise, mussels exposed to explosives vertically in 30 cm intervals in the same region showed high levels of explosives in their tissue (Fig. 9). While in an altitude of 10 cm from the ground level, TNT, 2-ADNT and 4-ADNT were found at levels of around 40, 50, and 145 ng/g mussel wet weight, respectively, the values decreased upward the water column (at 40 cm: around 30 ng/g of 2-ADNT and 60 ng/g of 4-ADNT; at 70 cm: around 30 ng/g of 2-ADNT and 50 ng/g of 4-ADNT; at 100 cm: around 10 ng/g of 2-ADNT and 20 ng/g of 4-ADNT). No TNT was detected in at 40 cm, 70 cm and 100 cm height.

### Risk assessment

TNT and its metabolites are known to be toxic and, even more, carcinogenic. Of major interest, therefore, was the question whether the consumption of blue mussels from the munitions dumpsite Kolberger Heide in Kiel Bight in the Baltic Sea was safe for human seafood consumers. TNT is currently classified by the “German MAK Commission” (MAK 2019) as belonging to Group 2 (“Substances that are considered to be carcinogenic in humans”). Because of the carcinogenicity of TNT and its metabolites with non-threshold effects, health risk assessment was performed here by using the margin of exposure (MOE) concept (EPA IRIS 1993). Moreover, since only two reliable studies on the carcinogenicity of TNT in animal studies are available (Furedi et al. 1984a, b), instead of the BMDL10 concept, the T25 method was applied to define the point of departure (POD) and to infer a possible health risk for the human seafood consumer (ECETOC Technical Report No. 83, 2002).

For calculating the carcinogenic risk, the following parameters were used: (1) the concentration of TNT and its metabolites 2-ADNT, 4-ADNT and 2,4-DA-6-NT were measured in the mussels; (2) the per capita consumption of fish or seafood in Germany of 39 g per day (FIZ 2017); and (3) the carcinogenicity of TNT determined from animal experiments (rats: 50 mg/kg b.w. per day; mouse: 1.5 mg/kg b.w. per day) (Furedi et al. 1984a, b). Since there are only limited or no data available on the carcinogenicity of 2-ADNT, 4-ADNT and 2,4-DA-6-NT, the risk related to dietary exposure to these compounds was assessed equal to the carcinogenicity for TNT and calculated as the sum of all these compounds. Details on the assessment of health risks associated with the toxicity and carcinogenicity of explosives such as TNT, RDX, HMX and metabolites thereof will be published in a special follow-up paper.

Overall, a preliminary toxicological risk assessment of the mussels contaminated with explosives from the different study sub-sites revealed two different scenarios: With regard to mussels transplanted directly at the corroding mines (study sub-site 1), the carcinogenic risk for the human seafood consumer was low, as the calculated MOE was higher than 25,000. In light of this MOE, consumption of these mussels was unlikely to be of health concern (of note: only 4-ADNT was detected).

In contrast, mussels transplanted in the direct vicinity to larger chunks or smaller pieces of free-lying explosives within or near craters (study sub-sites 2 and 3) contained significantly higher levels of TNT and 2-ADNT, 4-ADNT and 2,4-DA-6-NT metabolites. Here, calculated MOEs were lower than 25,000, indicating an individual cancer risk upon regular consumption of these mussels.

It should be emphasized here that the cancer risk for human seafood consumers was assessed using a worst-case scenario. First, all substances were set equally carcinogenic as the parent TNT. Second, an average daily intake of fish and marine food of 39 g (FIZ 2017) per person in Germany does not only consist of mussels, but also comprises fish, shrimp and others. Third, it was assumed that individuals consume mussels daily for their entire lifetime (70 years).

## Discussion

Since World War I, considerable amounts of warfare material have been dumped in the sea worldwide (Beddington and Kinloch 2005), but little is known about the fate of the explosive components in the marine environment. In an earlier study, we successfully transferred caging approaches with blue mussels (*Mytilus* spp.) (Strehse and Maser 2020) into an on-site biomonitoring system which can also be used for long-term studies to control the release of explosives from corroding munitions (Strehse et al. 2017). Indeed, with this system we were the first to show that these toxic explosive chemicals distribute in the marine environment and pose a threat to the marine ecosphere (Appel et al. 2018; Strehse et al. 2017). Here, we show that blast-in-place (BiP) operations lead to a considerably higher accumulation of TNT and its metabolic derivatives in the exposed mussels, thereby increasing the risk for environmental damage. A toxicological risk assessment from our data even led to the conclusion that these explosives may enter the marine food chain and affect human health upon consuming sea food.

Mussels are ideally suitable for controlled biomonitoring. They are filter feeding organisms thereby concentrating chemicals in their tissues, and they are robust and survive under moderate levels of different pollutions. Moreover, the blue mussel is an important seafood species and can thus simultaneously be used as an indicator for the entry of toxic substances into the marine food chain (Farrington et al. 1983).

Controlled blast-in-place (BiP) operations are still a common practice worldwide to prevent endangering today's shipping traffic or the installation of pipelines and offshore plants by uncontrolled and unforeseen explosions. However, BiP operations often result in incomplete (low-order) detonation, leaving substantial quantities of the explosive materials on the seafloor (Kalderis et al. 2011; NATO 2010).

Kolberger Heide in the Baltic near Kiel Fjord is a region of historical munitions disposal and is known to contain both German and British ordnance from World War II (Nehring 2008). Approximately 30,000 t of munitions including mines and depth charges were originally dumped in the area. Sub-sites within the Kolberger Heide represent intact and corroded munitions as well as completely exposed munition solids resulting from low-order detonation during BiP operations of in situ munitions disposal by the Explosive Ordnance Disposal of the Federal State of Schleswig–Holstein due to plans for re-routing a shipping lane (Koschinski and Kock 2009).

For our studies, we selected three study sub-sites within this area: study sub-site 1, where we transplanted mussels at six moorings around corroding, but sill encased mines; study sub-site 2 with one mooring directly at a chunk of unexploded hexanite; and study sub-site 3 where we exposed mussels on the seafloor covered with scattered smaller hexanite solids.

In mussels exposed directly to the corroding mines (study sub-site 1), we only detected 4-ADNT in relative low concentrations, ranging from 3.31 ± 1.18 to 4.94 ± 1.60 ng/g (mussel wet weight). The values did not differ significantly with regard to the distance to the mine mount or regarding the position of the mussels on the mooring (1 m above or directly on the ground) (Fig. 6), indicating that the leaking explosive chemicals have dissolved and are more or less evenly distributed in the water column around the mine pile. In neither mussel of the six moorings arranged around the mine mount, 2-ADNT, 2,4-DA-6-NT nor TNT itself could be detected.

It has been anticipated that very high and toxic concentrations may appear in the sediment and sediment pore water in the close vicinity of a munition fragment, but only a short distance away the concentrations may drop to non-toxic and non-detected levels (Beck et al. 2019; Rosen and Lotufo 2010). This may indeed be true for a complete and/or corroding encased mine as a point source of munitions chemicals. However, BiP operations that distribute multiple munitions material on the seabed may yield a completely different scenario.

In mussels from study sub-site 2 (directly transplanted in direct vicinity of a 50 cm diameter chunk of the explosive material hexanite close to a blast crater site, the TNT metabolites 2-ADNT, 4-ADNT and 2,4-DA-6-NT as well as TNT itself were found. Interestingly, the average concentrations were several-fold higher as compared to study sub-site 1: 2-ADNT = 103.75 ± 12.77 ng/g, 4-ADNT = 131.31 ± 9.53 ng/g, 2,4-DA-6-NT = 64.03 ± 7.34 ng/g and TNT = 31.04 ± 3.26 ng/g mussel wet weight, respectively. Obviously, free-lying explosive fragments resting on the seabed result in an increased entry and accumulation of explosive chemicals within the exposed mussels. In mussels of sub-site 2, placed 1 m above the ground, neither TNT nor 2-ADNT could be detected, but 4-ADNT and 2,4-DA-6-NT were determined with average concentrations of 8.71 ± 2.88 ng/g and 9.84 ± 0.15 ng/g mussel wet weight, respectively (Fig. 7). This obviously reflects the situation of study sub-site 1, in that also here the explosive material has evenly dissolved in the water column.

Water samples collected manually by scientific divers at close intervals very near and vertical to the exposed munitions surface yielded 3100 μg × l^−1^ dissolved TNT directly at a piece of solid hexanite, whereas concentrations declined rapidly away from the hexanite surface, to 16 μg × l^−1^ at a 1 cm distance and finally to 3.3 μg × l^−1^ at a 50 cm distance (Beck et al. 2019). This explains why the data derived from our studies generally showed elevated levels of explosive chemicals in mussels exposed near the munitions surface, while the concentrations decline upward of the water column.

Likewise, in study sub-site 3, in which mussels had been exposed to explosives in a region with scattered smaller hexanite solids on the seafloor, high concentrations of the explosives 2-ADNT, 4-ADNT and 2,4-DA-6-NT were determined (Fig. 8). While TNT itself was absent, the sum of all TNT metabolites in each individual mussel ranged from 125 to 270 ng/g mussel wet weight. This in turn reflects the situation of study sub-site 2, in that free-lying explosive fragments resting on the seabed result in an increased entry and accumulation of explosive chemicals within the exposed mussels.

The data derived from our vertical measurement approach also showed high levels near the munitions surface (at 10 cm height), with a decline upward of the water column (Fig. 9). This trend suggests that explosive chemicals are released to the water column by dissolution of the solids, while mixing processes dilute them within 1 m of the munitions surface. This is consistent with explosives gradients observed by Rosen and colleagues (Rosen et al. 2018) in an experiment using passive samplers and an explosive point source. Some variability was observed for samples being exposed horizontally in study sub-site 3, as this could mean that the mussels were obviously subject to small-scale spatial distribution of the hexanite.

Overall, these high concentrations of explosive chemicals within the mussel tissue could have arisen from direct contact of the mussels with explosive chunks or solids at the seafloor. This, in turn, means that the corrosive, but still present, metal shell of the munition body protects against an accumulation of the toxic and carcinogenic substances in the marine food web.

Leaching of explosive chemicals from the munitions, dissolution rates into the surrounding sediment and/or water column as well as entry and accumulation in the marine biota are subject to several variables/parameters (see below).

Munitions exposed to seawater for long periods of time experience extensive corrosion, which is governed by salinity, oxygen content, temperature, and the speed of water currents. The yearly estimated corrosion rate of steel in saline water is 0.01–0.575 mm (HELCOM, 1995; Russel et al. 2006; Sanderson et al. 2008). According to a Russian estimate, corrosion will lead to a maximum chemical release rates in the early twenty-first century from submerged munitions in the Baltic Sea (Glasby 1997; Granbom 1994; Malyshev 1996).

Dissolution of chemicals from explosive solids represents the initial controlling factor for release into the environment. Dissolution rates of explosive materials range typically between 0.5 and 50 mg × cm^−2^ day^−1^ (Beck et al. 2019). Beck et al. (2019) argued that the dissolution rate under laboratory conditions increases rapidly with temperature, is slightly reduced in seawater compared with freshwater, depends on the formulation and is highly effected by stirring or mixing speed. Hence, dissolution rates in the natural marine environment depend on temperature, currents, tidal ranges and ionic strength (salinity) and reliably determine the transport and fate of these compounds in the water column. Moreover, the total release of toxic explosive chemicals from underwater munitions depends on the surface area of the material which is a critical factor for risk assessments. It is therefore an urgent requirement to refrain from BIPs if they are not absolutely necessary due to security concerns, as BiP operations may result in scattering of smaller and larger solids of explosive materials on the seafloor.

Overall, these variables make it clear that to thoroughly determine the scale and extent of explosive chemicals spread in marine systems, sophisticated long-term biomonitoring strategies in free field studies like that of our approach with blue mussels are necessary. The collected data will reflect the state of contamination in this area and will then make the basis for further toxicological risk assessment.

Several studies have demonstrated the toxicity of energetic compounds on aquatic organisms (Juhasz and Naidu 2007; Lotufo et al. 2013; Talmage et al. 1999). Sub-lethal response to TNT exposure has been reported and includes effects on growth, reproduction, germination, gene transcription as well as on nervous, immune and blood systems (Gong et al. 2007). There is some evidence that the TNT metabolites (i.e., 2-ADNT, 4-ADNT and 2,4-DA-6-NT) may be more toxic than the parent TNT, but reliable data on these compounds are limited or lacking for the marine environment (Lotufo et al. 2013).

Transformation processes of parent TNT include photolysis, hydrolysis, oxidation, reduction, and biological transformation (Goodfellow et al. 1983). 4-ADNT appeared to be the most abundant explosive compound found in all samples of the present study. The presence of low levels or even the absence of TNT itself throughout our study area was likely a consequence of microbial metabolism of TNT in the sediment or conversion by mussel enzymes to 2-ADNT, 4-ADNT and 2,4-DA-6-NT (Hawari et al. 2000).

While sediment living organisms are obviously at the highest risk of being exposed to harmful concentrations of munitions residues, discarded military munitions and unexploded ordnance can effectively serve as seafloor habitat for marine organisms. This may lead to even higher exposure scenarios, especially at BiP sites, such that these organisms are exposed to concentrations many orders of magnitude higher than pelagic organisms.

Upon entry into the marine food chain, explosive chemicals represent a direct hazard to humans through seafood consumption, because these substances are cytotoxic, genotoxic, and carcinogenic (Bolt et al. 2006; Koske et al. 2019; Lotufo et al. 2013). The mechanism by which TNT and its metabolites exert toxic effects on a large number of organs has not been fully elucidated. With chronic occupational exposure, typical effects were methemoglobin formation up to cyanosis, anemia, damage to bone marrow and spleen, cataract formation (TNT star), dermatitis, hepatitis and toxic polyneuritis. Damage to the hematopoietic system and the liver was also found in animals (Bolt et al. 2006). US EPA set a reference dose at 0.5 μg/kg bw × day based on a study on dogs exposed for 26 weeks, considering the hepatotoxic effects as the critical effect (US-EPA, 1988).

The genotoxicity of TNT in bacteria has been confirmed both with and without metabolic activation, but contradictory results have been obtained in mammalian cells in vitro (Bolt et al. 2006). TNT has been tested for carcinogenicity in 2-year bioassays in rats and mice (Furedi et al. 1984a, b). After administration of TNT via diet, carcinoma of the urinary bladder and hepatocellular neoplasms were observed in the rat (Furedi et al. 1984a), while malignant lymphoma combined with lymphocytic and granulocytic leukemia in the spleen significantly increased in mice (Furedi et al. 1984b). US-EPA (EPA IRIS 1993) concluded that TNT is a possible human carcinogen (Class C). A study on human workers found elevated levels of chromosomal aberrations in a subset of TNT-exposed workers who were positive for *N*-acetyltransferase (*NAT1*) (rapid acetylator), with the null glutathione-*S*-transferase (GST) T1 (*GSTT1*) or *GSTM1* genotype (Sabbioni et al. 2007). In Germany, TNT has been classified as belonging to group 2 (“Substances that are considered to be carcinogenic in humans”) (MAK 2019).

In general, health risk assessments for carcinogenic compounds with non-threshold effects are performed by using the margin of exposure (MOE) concept (EPA 2012). Since only two reliable studies on the carcinogenicity of TNT in animal studies are available (Furedi et al. 1984a, b), instead of the BMDL10 concept the T25 method (ECETOC Technical Report No. 83 2002) was applied here to define the point of departure (POD) and to infer a possible health risk for the human seafood consumer.

Human exposure was assessed based on national diet studies on fish consumption in Germany (FIZ 2017). Overall, the toxicological risk assessment of the mussels contaminated with explosive chemicals from the different study sub-sites revealed two different scenarios: With regard to mussels transplanted directly at the corroding mines (study sub-site 1), the carcinogenic risk for the human seafood consumer was low, as the calculated MOE was higher than 25,000. In light of this MOE, consumption of these mussels was unlikely to be of health concern. In contrast, mussels transplanted in direct vicinity to larger chunks or smaller pieces of free-lying explosive materials within or near craters (study sub-sites 2 and 3) contained significantly higher levels of TNT and 2-ADNT, 4-ADNT and 2,4DA-6-NT metabolites. Here, calculated MOEs were lower than 25,000, indicating an individual cancer risk upon regular consumption of these mussels.

Of note, continuous biomonitoring followed by toxicological risk assessment for the human seafood consumer may generally be important in an area with nearby fishing activities or aquatic industry, e.g., when fish farms are located in the vicinity of sea dumped munitions sites or even at BiP sites. This fish could accumulate explosive chemicals in an extent that would be unacceptable for the consumer. Although the results from the mussel samples do not give a direct answer to the question whether the fish is consumable or not, it will provide a warning system that the fish farming industry should undertake respective studies on their fish population.

## Conclusion

We have shown that the application of the blue mussel (*Mytilus* spp.) as a biomonitoring system in free field studies together with high-resolution GC/MS–MS laboratory analysis represent a sensitive and reliable indicator for the pollution of the marine environment by chemicals released from munitions. While direct measurement of explosives in seawater or sediments may be subject to various disturbance variables (e.g., currents, salinity and temperature), our biomonitoring system with blue mussels may offer a more accurate assessment and greater reliability, as the determination of explosive chemicals at environmental concentrations covers a larger time frame. As such, this kind of biomonitoring system not only allows discriminating between no-measures sites and sites that need to be investigated further, but could also serve as an early warning system for a munitions dumping ground. Moreover, mussels are an important seafood species such that data derived thereof could help to assess risks associated with the marine food chain and the human seafood consumer.

The explosive materials that were fully encased for decades have been started to leak out and threaten marine waters and ecosystems, a problem that is further exacerbated by BiPs. The high concentrations of explosive chemicals detected in the mussel tissues at the crater site could be linked to blast-in-place operations for the purpose of munitions disposal, which obviously resulted in chunks of unexploded residuals on the seafloor, resulting in an increased environmental exposure of benthic organisms to explosive chemicals.
